# Progressively Increased M50 Responses to Repeated Sounds in Autism Spectrum Disorder with Auditory Hypersensitivity: A Magnetoencephalographic Study

**DOI:** 10.1371/journal.pone.0102599

**Published:** 2014-07-23

**Authors:** Junko Matsuzaki, Kuriko Kagitani-Shimono, Hisato Sugata, Masayuki Hirata, Ryuzo Hanaie, Fumiyo Nagatani, Masaya Tachibana, Koji Tominaga, Ikuko Mohri, Masako Taniike

**Affiliations:** 1 Molecular Research Center for Children’s Mental Development, United Graduate School of Child Development, Osaka University Graduate School of Medicine, Osaka, Japan; 2 United Graduate School of Child Development, Osaka University Graduate School of Medicine, Osaka, Japan; 3 Department of Pediatrics, Osaka University Graduate School of Medicine, Osaka, Japan; 4 Department of Neurosurgery, Osaka University Graduate School of Medicine, Osaka, Japan; Vanderbilt University, United States of America

## Abstract

The aim of this study was to investigate the differential time-course responses of the auditory cortex to repeated auditory stimuli in children with autism spectrum disorder (ASD) showing auditory hypersensitivity. Auditory-evoked field values were obtained from 21 boys with ASD (12 with and 9 without auditory hypersensitivity) and 15 age-matched typically developing controls. M50 dipole moments were significantly increased during the time-course study only in the ASD with auditory hypersensitivity compared with those for the other two groups. The boys having ASD with auditory hypersensitivity also showed more prolonged response duration than those in the other two groups. The response duration was significantly related to the severity of auditory hypersensitivity. We propose that auditory hypersensitivity is associated with decreased inhibitory processing, possibly resulting from an abnormal sensory gating system or dysfunction of inhibitory interneurons.

## Introduction

Autism spectrum disorder (ASD) is a neurodevelopmental disorder characterized by qualitative impairments in social interaction and communication, as well as by restricted behavior and interests [1]. In the Diagnostic and Statistical Manual of Mental Disorders (5th edition), in addition to those classical features, sensory abnormalities were appended to the diagnostic criteria for ASD [2]. Unusual perceptual abilities are also common in ASD; for example, many individuals with ASD express extreme reactions to at least one sensory modality, such as auditory stimulation [3]. These individuals perceive innocuous sounds as painful and frightening noise; and in some cases, those sounds may be perceived as phobic stimuli and result in radical behavioral responses in autism spectrum individuals. There are very few clinical interventions available for the treatment of auditory hypersensitivity; and, also, the underlying physiological mechanism has yet to be elucidated. We recently reported that auditory hypersensitivity in ASD is strongly correlated with delayed M50/M100 peak latencies as well as with increased M50 dipole moments [4]. M50/M100 peak responses are activated by auditory stimuli, which responses reflect the process of sensory input and originate in or near the primary auditory cortex [5]. We concluded that these phenomena possibly resulted from neurological immaturity or functional abnormalities in the primary auditory cortex.

On the other hand, Guiraud *et al*. [6] reported that infants at high risk for autism show less habituation to repeated sounds compared with those at low risk; and they speculated that this reduced habituation could be associated with hyposensitivity or over-reactivity to repeated stimulation. Interestingly, in patients having schizophrenia with sensory abnormalities, P50 event-related potential responses are not diminished by repeated sound stimuli [7]. In order to clarify the differential time-course responses of the primary auditory cortex to repeated sounds, we examined M50/M100 responses in individuals with ASD and analyzed these responses in relation to those in patients with characteristic traits of auditory hypersensitivity.

## Methods

### Participants

Twenty-one male children diagnosed with ASD and 15 age-matched typically developing control males (TD) were recruited at the Osaka University Hospital (Table 1). The diagnosis for ASD was made according to the Diagnostic and Statistical Manual of Mental Disorders (4^th^ edition) text revision (DSM-IV-TR) [1] and was made by an experienced clinician. Furthermore, the diagnosis was confirmed by the Japanese version of the Autism Screening Questionnaire (ASQ - J) [8], [9] and Autism Diagnostic Observation Schedule - Generic (ADOS-G) [10]. The intelligence quotient (IQ) was assessed by the Wechsler Intelligence Scale for Children, 3rd version (WISC-III). We assessed the auditory hypersensitivity and behavioral problems by using the Sensory Profile (SP) [11] and the Japanese version of the Child Behavior Checklist (CBCL), respectively [12], [13]. Children with ASD were divided into two groups based on the auditory item score of their SP: ASD individuals with auditory hypersensitivity were defined as those having a low SP score of under 30. TD participants were age-matched children without a history of neurological diseases or developmental disorders, and who also were not receiving special education services. In addition, all TD individuals were assessed by the ASQ-J and SP, and they showed no autism traits or sensory problems. Their hearing ability was normal according to available medical records or parent’s report. Written informed consent was obtained from the parents of all participants in the study, which was approved by the Institutional Review Board of Osaka University Hospital.

**Table 1:** Demographic Information.

Groups	TD(N = 15)	ASD without auditory Hypersensitivity(N = 9)	ASD with auditory hypersensitivity(N = 12)
	Mean±SD	Mean±SD	Mean±SD
Age(years)	9.80±1.66	9.40±1.64	9.48±1.70
FIQ	–	99.22±11.38	97.58±18.06
ASQ	2.60±3.02^*1,*2^	15.11±4.73^*1^	14.58±6.05*^2^
SP score	38.73±1.83	31.78±2.44	21.67±5.60
CBCL	–	51.22±13.08	63.38±20.58

FIQ: Full-scale intelligence quotient of Wechsler Intelligence Scale for Children, 3rd version, ASQ: Autism Screening Questionnaire (cut-off ≧ 13). SP: Sensory Profile (cut-off ≧ 30). CBCL: Child Behavior Checklist.

For the ASQ, there were significant differences between TD and ASD with hypersensitivity (*1; *p*<0.01) and also between TD and ASD without hypersensitivity (*2; *p*<0.01).

There were significant differences in SP between TD and both ASD without/with hypersensitivity and also between ASD without and with hypersensitivity (all *; *p*<0.01).

### Auditory Stimuli

Auditory stimuli, which were made by a presentation system (Presentation, Neurobehavioral System, Inc. San Francisco, CA), were delivered via a sound pressure transducer and sound conduction tubing to the participant’s auditory canal via ear tip inserts. A 1,000- Hz sinusoidal tone pip of 200 msec’ duration was binaurally presented (80 dB, with 10-msec rise and fall times). Stimuli were randomly presented with inter-stimulus intervals of 2,700 to 3,300 msec. The total number of stimuli was 100.

### Measurements

Before recording by magnetoencephalography (MEG), we scanned the 3D facial surface of each participant (Fast SCAN Cobra, POLHEMUS, ARANTZ Scanning Limited, Christchurch, New Zealand), together with the five head-marker coils as fiduciary points (the external meatus of each ear, two points on the forehead, and nasion).

While lying down on a bed in a magnetically-shielded room, the participants were examined by a 160-channel whole-head MEG system equipped with SQUID gradiometers (PQ1160C, Yokogawa Electric Corporation, Tokyo, Japan). The positions of head marker coils were obtained before and after recording to evaluate head movement [14]. Data were acquired at a sampling rate of 1000 Hz, with an online band pass filter between 0.03 Hz and 200 Hz.

Individual anatomical magnetic resonance image (MRI) data were obtained with a 3.0-Tesla whole-body magnetic resonance scanner equipped with a standard whole-head coil (Signa Excite HD; GE Healthcare, Milwaukee, USA). A 3D-T1-weighted axial protocol was used. Imaging parameters for 3D-T1-weighted images were as follow: 3D-spoiled GRASS sequence, repetition time (TR) = 10.1 ms; echo time (TE) = 3.0 ms; flip angle = 18°; field of view (FOV) = 220×220 mm2; matrix size = 320×256; slice thickness = 1.4 mm; number of excitations (NEX) = 1.A 3D-T1 [15]. 3D facial surface data and fiduciary points were superimposed on the individual MRI data. The MEG data were superimposed on the individual MRI with an anatomical accuracy of a few millimeters.

### Data analysis

**Figure 1:**
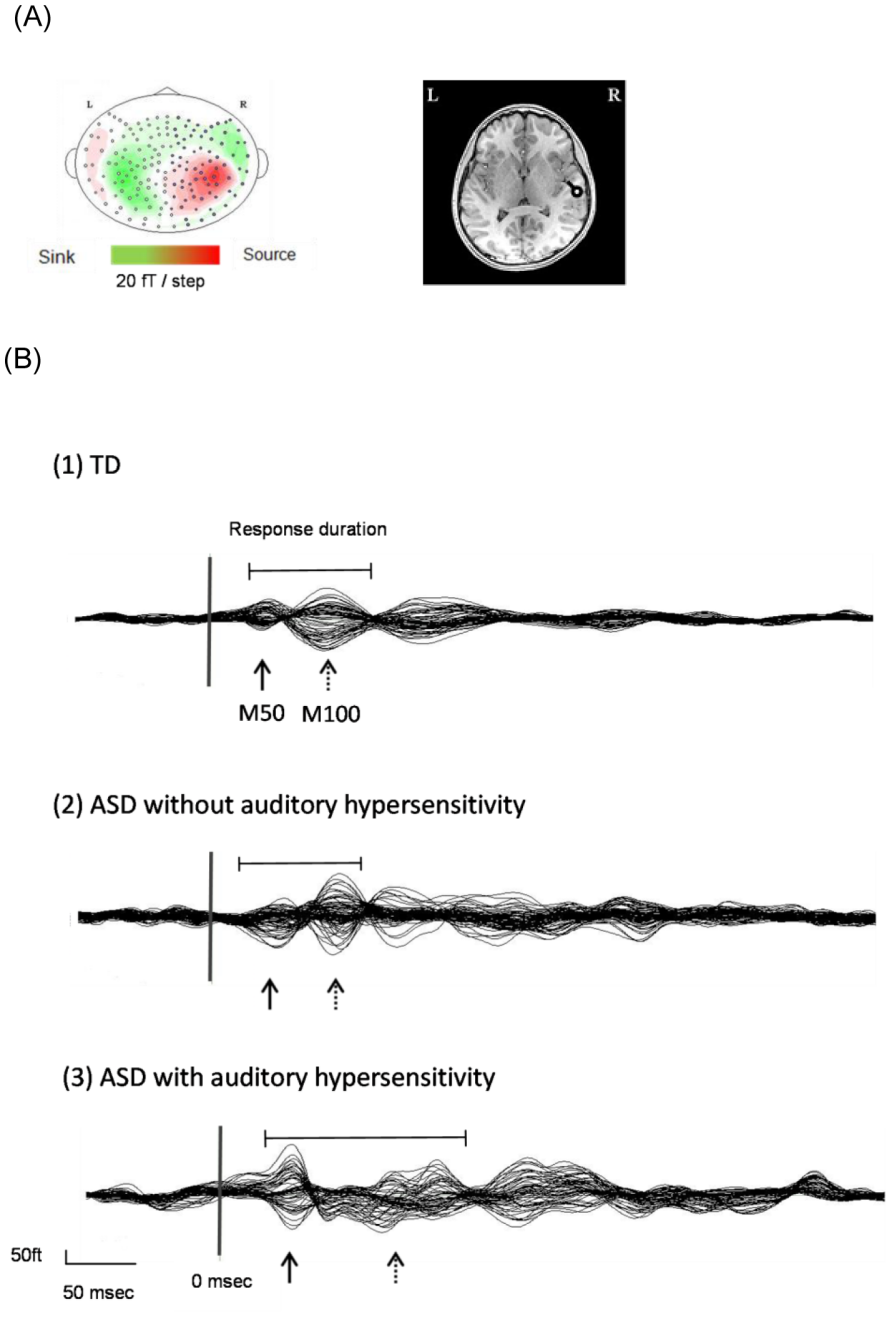
Magnetic isofield maps (A-left), and dipole sources (A-right), which were superimposed on the MRI of individual subjects, were selected; and examples from a TD individual are shown. M50, M100 average waveforms as examples from each of the 3 groups were selected and are shown (B). Vertical lines on the averaged waveform trace indicate stimulus onset (0 msec). Arrows point to M50; and dashed arrows, to M100. The response duration of early auditory processing was determined as the interval between the rising point of the M50 and the returning point to baseline of the M100.

The MEG data were analyzed by using standard analysis software (MEG Laboratory, Yokogawa Electric Corporation, Tokyo, Japan). The epochs were defined from 100 msec before stimulation to 1000 msec after it. This pre-stimulus period of 100 msec was used as the baseline for determination of ambient brain activity and noise of each epoch. Before analysis, all epochs for each participant were confirmed not to have included artifacts such as eye blinks or, head movements. To determine the M50/M100 peak, we calculated the root mean square (RMS) of half of all channels. The M50/M100 peak was determined as the peak in RMS value in the interval of 30–70 ms and 80–200 ms, respectively. The response duration of early auditory processing was determined as the interval between rising point of the M50 and the returning point to baseline of M100 (Fig. 1-B). For the time-course analysis of the M50/M100 component, we divided 100 epochs into five successive periods (a; 1–33, b; 15–47, c; 34–66, d; 48–80, e; 67–100), and analyzed the dipole moment and latency in each part. We calculated dipole moments (intensity) of the magnetic source by using the equivalent current dipole (ECD) estimation method [16]. Also, we accepted only those estimated ECDs that showed a goodness of fit (GOF) of over 80% [17]. The waveforms averaged in each epoch were high pass-filtered by using a cut-off frequency of 3 Hz and low pass-filtered by using one of 45 Hz. We evaluated responses of each hemisphere by using half of all channels.

All data analyses were performed by using SPSS statistics for Windows software, version 22.0 (IBM, Tokyo, Japan), and the level of significance was set at *p*<0.05. One-way analysis of variance (ANOVA) was used to compare age, IQ, SP, CBCL scores, and response duration. For M50/M100 measurements, we used a repeated measures linear model with five successive periods (a/b/c/d/e) as within-subjects factor and the 3 different groups (TD/ASD without auditory hypersensitivity/ASD with auditory hypersensitivity) as between-subjects factor. We tested data for normality by using the Kolmogorov-Smirnov test. The Greenhouse-Geisser correction was applied to the data, if the Mauchly’s test of sphericity was statistically significant. Bonferroni correction was applied for multiple comparison analysis. The Pearson test was used to examine the correlation.

## Results

### Demographics

There was no main effect of age [*F* (2, 33) = 0.202, *p* = 0.820, *η* ^2^ = .01]. In ASQ scores, there was a significant main effect of group [*F* (2, 33) = 30.372, *p*<0.001, *η* ^2^ = .65]. The ASQ scores of ASD with/without auditory hypersensitivity were significantly higher than those of the TD (*p*<0.001). The auditory score of the SP revealed a significant main effect of group [*F* (2, 33) = 73. 058, *p*<0.001, *η* ^2^ = .82]. The ASD with auditory hypersensitivity group showed lower SP scores than those of the ASD without auditory hypersensitivity and TD groups [*p*<0.001, Table 1]. The CBCL scores of the patients having ASD with hypersensitivity were higher than those of the ones having ASD without hypersensitivity; however, the difference was not significant [*F* (1, 19) = 2.510, *p* = 0.130, *η* ^2^ = .12]. The scores of full intelligence quotient showed no difference between ASD groups with and without auditory hypersensitivity [*F* (1, 19) = 0.057, *p* = 0.814, *η* ^2^ = .02].

### M50/M100 dipole moments

Magnetic isofield maps (Fig. 1-A-left) and dipole sources (Fig. 1-A-right), which were superimposed on the MRI of individual subjects, were prepared; and examples from a TD individual are shown (Fig. 1-A). M50, M100 average waveforms were selected as an example from each of the 3 groups (Fig. 1-B).

**Figure 2:**
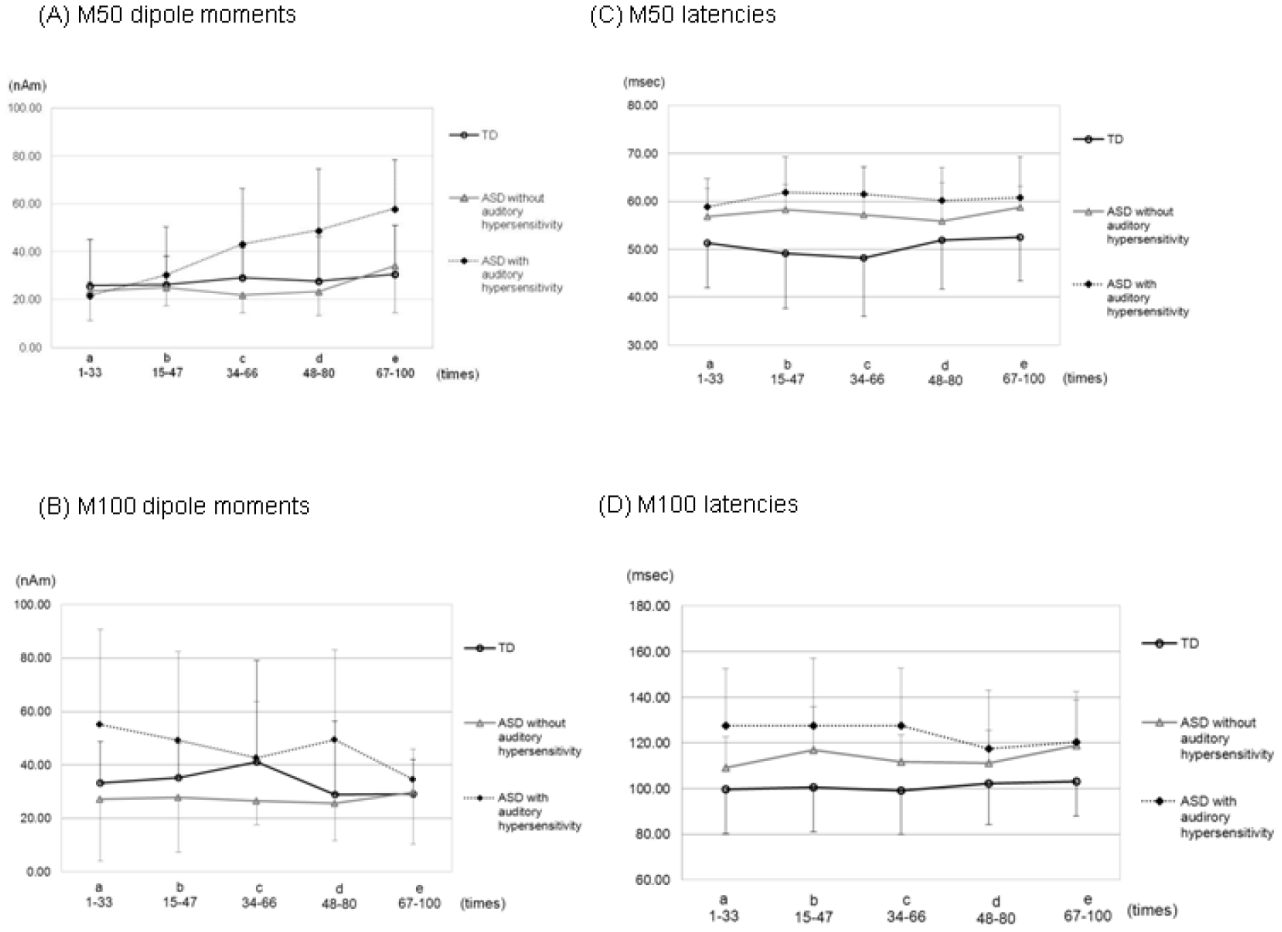
Data were divided into 5 successive periods consisting of a total of 100 epochs (a; 1–33, b; 15–47, c; 34–66, d; 48–80, e; 67–100). Error bars represent 1 standard error of the mean. A) Mean M50 dipole moments. The ASD with auditory hypersensitivity group showed a significant increase in the M50 dipole moment with time (*p*<0.01). B) Mean M100 dipole moments. The 3 groups did not show any significant differences among them in any of the successive periods. C) Mean M50 latencies. There were no significant differences among the groups in any of the successive periods. D) Mean M100 latencies. Again, no differences were noted.

As shown in Fig. 2-A, significant interactions between group and periods were observed for M50 dipole moments [*F* (8,132) = 3.101, *p* = 0.003, *η* ^2^ = .08]. In addition, there were significant main effects of group [*F* (2, 33) = 6.395, *p* = 0.004, *η* ^2^ = .11] and periods [*F* (4,132) = 6.842, *p*<0.001, *η* ^2^ = .08]. From the simple effect analysis, we found significant differences between part a and b (*p* = 0.022), a and d (*p* = 0.001), a and e (*p*<0.001), and also b and e (*p* = 0.001) in the ASD with auditory hypersensitivity group. The M50 dipole moment showed a significant increase with time only in this group. In addition, there was a significant difference in part c (*p* = 0.014) between the ASD with auditory hypersensitivity group and ASD without auditory hypersensitivity one. There was a significant difference in part d between ASD with auditory hypersensitivity group and ASD without auditory hypersensitivity group (*p* = 0.002), and between ASD with auditory hypersensitivity group and TD group (*p* = 0.004). Also, in the case of part e, there was a significant difference between ASD with auditory hypersensitivity group and ASD without auditory hypersensitivity group (*p* = 0.005), and between ASD with auditory hypersensitivity group and TD group (*p*<0.001). The ASD with auditory hypersensitivity group showed significantly larger dipole moments than the other groups.

As was shown in Fig. 2-B, there were no significant interactions between groups and periods for M100 dipole moments [*F* (4.11, 67.816) = 0.882, *p* = 0.482, *η* ^2^ = .03]. Also, there was no significant main effect of periods [*F* (2.055, 67.816) = 1.023, *p* = 0.367, *η* ^2^ = .02]. In addition, there was a significant main effect of groups [*F* (2, 33) = 3.524, *p* = 0.041, *η* ^2^ = .08]. However, from the multiple comparison analysis, there were no significant differences between groups in any periods.

### M50/M100 latencies

As shown in Fig. 2-C, there were no significant interactions between group and periods [*F* (8,132) = 0.276, *p* = 0.973, *η* ^2^ = .01] for M50 latencies. Also, there was no significant main effect of periods [*F* (4,132) = 0.999, *p* = 0.410, *η* ^2^ = .01]. However, there was a significant main effect of group [*F* (2, 33) = 7.418, *p* = 0.002, *η* ^2^ = .21]. From the multiple comparison analysis, M50 latencies were significantly delayed in the ASD with auditory hypersensitivity group than in the TD group in all periods (*p*<0.03).

As was shown in Fig. 2-D, there were no significant interactions between groups and periods for M100 latencies [*F* (5.102, 84.175) = 1.186, *p* = 0.323, *η* ^2^ = .02]. Also, there was no significant main effect of periods [*F* (2.551, 84.175) = 0.683, *p* = 0.542, *η* ^2^ = .01]. On the other hand, there was a significant main effect of groups [*F* (2, 33) = 6.597, *p* = 0.004, *η* ^2^ = .21]. From the multiple comparison analysis, there were significant differences between ASD with auditory hypersensitivity group and TD group for periods a, b, and c (*p*<0.03). The ASD with auditory hypersensitivity group showed delayed M100 latencies compared with those for the TD group. Also, the ASD with auditory hypersensitivity group showed latencies that were more delayed than the ASD without auditory hypersensitivity group; however, there were no statistically significant differences between these two groups.

### Response duration

**Figure 3:**
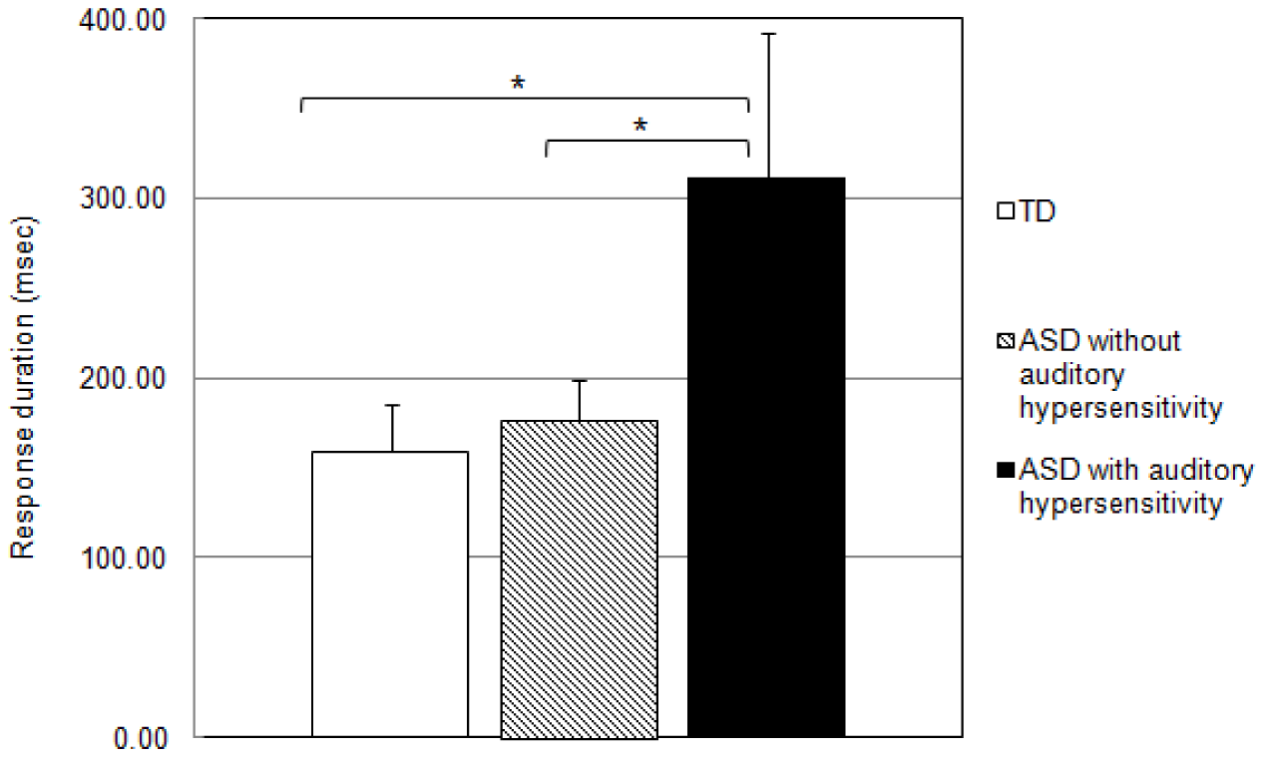
Mean response duration of each group. Error bars represent 1 standard error of the mean. The response duration in the ASD with auditory hypersensitivity group was significantly longer than that in the other 2 groups (*p*<0.05).

**Figure 4:**
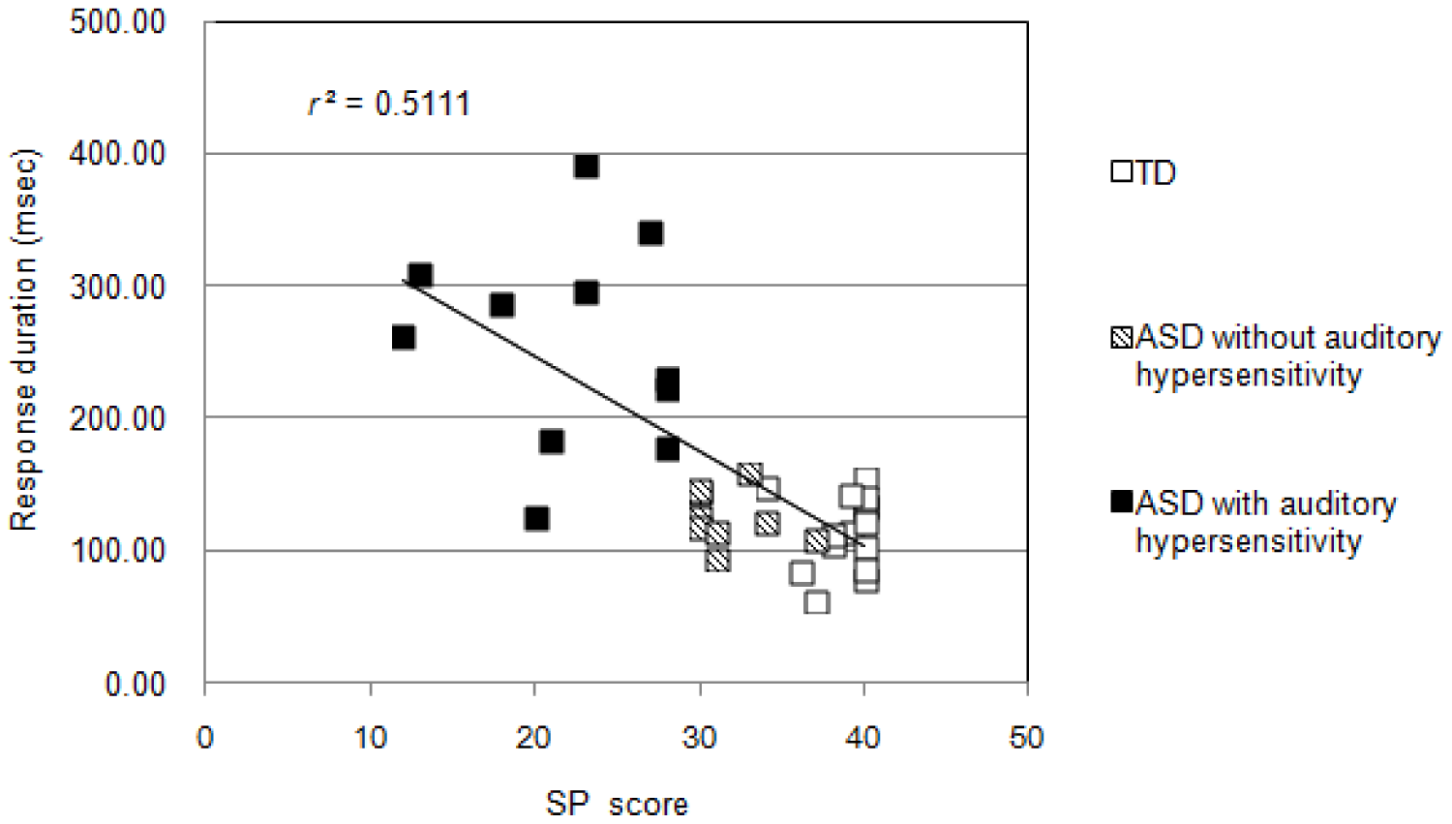
Scatter plot of response duration and auditory item scores of SP. Response duration was negatively correlated with auditory item scores of SP (*p*<0.05).

One-way ANOVA revealed a significant main effect of group for response duration [*F* (2, 32) = 32.84, *p*<0.001, *η* ^2^ = .67; Fig. 3]. From the multiple comparison analysis, individuals having ASD with auditory hypersensitivity showed a longer response duration than those in the other two groups (*p*<0.001). In addition, an obvious negative correlation was recognized between response duration and SP score (*r* = −0.747, *p*<0.001; Fig. 4). Also, there was positive correlation between response duration and CBCL score (*r* = 0.522, *p* = 0.183). There was no statistically significant correlation between response duration and FIQ score (*r* = 0.1424, *p* = 0.549).

## Discussion

The results of our present study showed differential time-course responses of the auditory cortex to repeated auditory stimuli in individuals having ASD with auditory hypersensitivity compared with those responses in TD subjects or those having ASD without hypersensitivity. The main findings of our study were the following: 1) The ASD with auditory hypersensitivity group showed increased M50 dipole moments over time. 2) Their response duration was significantly longer than that of the other two groups; and this prolonged response duration also significantly correlated negatively with their auditory scores of SP (i.e., severity of hypersensitivity). 3) In all groups, responses of M50/M100 latencies were constant with time.

In our previous study, we reported delayed M50 and M100 latencies in ASD with auditory hypersensitivity. These results may have been due to abnormalities in the myelination process, thus resulting in slower transmission rates in the central auditory pathways [4], [18]. In this present study, we found increased M50 dipole moments with time. However, there were no specific changes in M50 or M100 latency or in M100 dipole moments with time, in all groups.

Additionally, we found a significantly prolonged response duration in the ASD with auditory hypersensitivity group compared with that in the ASD without it or in the TD group. This prolonged response suggested the prolongation of convergence time, and reflected restoration of responses in the primary auditory cortex. In this study, we found that ASD with auditory hypersensitivity showed not only delayed M50/M100 peak latency, but also prolonged convergence time. Furthermore, this extension of the response duration correlated with the severity of auditory hypersensitivity. On the ground of the prolonged response duration, we speculate that the increased excitation and/or reduced inhibition might reflect hyperactivity in the primary auditory cortex, resulting in overlapped responses with time or increased M50 dipole moment responses in the ASD with auditory hypersensitivity group.

Recently, several studies have reported an aberrant white matter microstructure in ASD as compared with that in the controls [19], [20]. As described previously, we suggest that those phenomena may be associated with slowed and/or prolonged activity of the neural circuitry of the auditory cortex, resulting from its abnormal maturation in ASD with auditory hypersensitivity.

Alternatively, Rotschafer and Razak reported that a mouse model of Fragile X Syndrome (FXS), which exhibits autistic features, shows expanded frequency tuning, enhanced response magnitude, and more variable first spike latencies compared to wild-type controls [21]. They concluded that these results may reflect the auditory processing deficits seen in FXS patients [21]. FXS patients are known to show autistic symptoms and also auditory abnormalities. We speculate that those phenomena may be related to the characteristics of auditory hypersensitivity in ASD.

On the other hand, according to a pathological study on autistic postmortem brain, the minicolumns in auditory cortex revealed less peripheral neuropil space, which is the conduit for the GABAergic inhibitory local circuit projection [22], leading to the speculation that abnormalities of GABAergic interneurons might induce cortical hypersensitivity in the auditory cortex.

As another possible neurochemical pathophysiology of auditory hypersensitivity, Curtin et al. [23] reported that the serotonin 5-HT5A receptor regulates excitability in the auditory startle circuit in the pre-pulse inhibition (PPI) paradigm. Serotonin 5-HT5A contributes to sensorimotor integration and decision-making by directly regulating excitability. Moreover, Medan and Preuss [24] found that dopaminergic modulation regulates the time course and magnitude of PPI, whereas 5-HT regulates tonic excitability. Many studies have found abnormalities in the serotonin/dopamine system in autism patients [25], [26]. Therefore, we hypothesize that the auditory hypersensitivity in ASD could be related to dysfunctional regulation of the auditory startle circuit, a dysfunction resulting from abnormalities of serotonin 5-HT5A receptors or dopaminergic modulation.

Recently, some researchers have reported abnormal P50/M50 responses in the paired-click paradigm in patients with schizophrenia and other disorders, such as post-traumatic stress disorder [27], [28], which is associated with thalamic sensory gating system [7]. Also, the similarities between schizophrenia and autism both in genetic and behavioral characteristics have also been reported [29]–[31]. Therefore, we speculate that the increased M50 dipole moment seen in ASD with auditory hypersensitivity may be related to impairment of the sensory filter mechanism resulting from abnormalities of the thalamic sensory gating system. Meanwhile, M100 dipole moment did not change with time in any of the groups. Although the M50/M100 responses arise from both the auditory cortex, their functional role might be different [5].

On the other hand, latencies were constant with time in all groups. We have already reported a delayed M50/M100 peak latency in ASD with auditory hypersensitivity. Therefore, delayed M50/M100 latencies resulting from abnormalities in the myelination processes might be unaffected by the stimulus frequency.

Based on the findings of this present study, we suggest that characteristics of auditory hypersensitivity may be associated with abnormalities in the early stages of auditory processing and that it involves multiple neural mechanisms.

Limitations of this study are as follow: 1) We focused on responses of the auditory cortex. It remains unclear whether any specific activation induced by sound occurred in any other brain region. 2) From the result showing an increased M50 dipole moment, we speculated that the abnormalities such as increased excitation/reduced inhibition and the thalamic sensory gating system were involved. However, in this study, we did not use the paired-click paradigm. Thus, future work will be needed to determine the validity of this speculation. 3) We have supposed that our results may be related to auditory hypersensitivity behavior in actual life. However, we used only a sinusoidal tone pip as auditory stimuli. Therefore, further work will be needed by using real-life sounds (e.g., baby crying, hair dryer, etc).

Even though auditory hypersensitivity tends toward maladaptation of patients with ASD, there is no effective therapeutic approach yet. At least, our study has provided objective assessments of clinical symptoms and also beneficial information for understanding the characteristics of ASD patients and for supporting their quality of life.

## Conclusions

An increase in the M50 dipole moment with time and a prolonged response duration of the auditory cortex were found in our subjects having ASD with auditory hypersensitivity. In addition to the possibility of abnormalities of the maturational process, these phenomena might have occurred due to decreased inhibitory processing and an increase in the ratio of cortical excitation to inhibition, possibly resulting from an abnormal sensory gating system or dysfunction of inhibitory interneurons. Our findings suggest that auditory hypersensitivity may be associated with abnormalities in the early stages of auditory processing and that it involves multiple neural mechanisms. Understanding of the neurological basis of auditory hypersensitivity could have clinical benefits, resulting from validation of effective treatment for it in the future.
